# Interleukin-6 as inflammatory marker referring to multiple organ dysfunction syndrome in severely injured children

**DOI:** 10.1186/1757-7241-22-16

**Published:** 2014-03-03

**Authors:** Hagen Andruszkow, Janika Fischer, Michael Sasse, Ulf Brunnemer, Julia Helga Karla Andruszkow, Axel Gänsslen, Frank Hildebrand, Michael Frink

**Affiliations:** 1 Department of Orthopaedic Trauma at Aachen University, Pauwelsstraße 30, 52074 Aachen, Germany; 2 Center of Pediatric Surgery Hannover, Hannover Medical School and Bult Children’s Hospital, Carl Neuberg-Str. 1, 30625 Hannover, Germany; 3 Department for Trauma, Hand and Reconstructive Surgery, Medical Center Wolfsburg, Sauerbruchstr 7, 38440 Wolfsburg, Germany; 4 Department for Trauma, Hand and Reconstructive Surgery, University Medical Center Marburg, Baldingerstr, 35043 Marburg, Germany; 5 Trauma Department, Hannover Medical School, Carl Neuberg-Str. 1, 30625 Hannover, Germany; 6 Institute of Pathology, University Hospital Aachen, Pauwelsstraße 30, 52074 Aachen, Germany; 7 Clinic for Paediatric Cardiology and Internal Medicine, Carl Neuberg-Str. 1, 30625 Hannover, Germany

**Keywords:** Multiple trauma, Pediatric trauma, Multiple organ dysfunction syndrome, Multiple organ failure, MODS, Inflammatory response, Interleukin-6, IL-6

## Abstract

**Background:**

Despite the suggestion that the inflammatory response in traumatized children is functionally unique, prognostic markers predicting pediatric multiple organ failure are lacking. We intended to verify whether Interleukin-6 (IL-6) displays a pivotal role in pediatric trauma similar to adults.

**Methods:**

Traumatized children less than 18 years of age with an Injury Severity Score >9 points and consecutive admission to the hospital’s pediatric intensive care unit were included. Organ function was evaluated according to the score by Marshall et al. while IL-6 levels were measured repetitively every morning.

**Results:**

59 traumatized children were included (8.4 ± 4.4 years; 57.6% male gender). Incidence of MODS was 11.9%. No differences were found referring to age, gender, injury distribution or overall injury severity between children with and without MODS. Increased IL-6 levels during hospital admission were associated with injury severity (Spearman correlation: r = 0.522, p < 0.001), while an inconsistent association towards the development of MODS was proven at that time point (Spearman correlation: r = 0.180, p = 0.231; Pearson's correlation: r = 0.297, p = 0.045). However, increased IL-6 levels during the first two days were no longer associated with the injury severity but a significant correlation to MODS was measured.

**Conclusions:**

The presented prospective study is the first providing evidence for a correlation of IL-6 levels with injury severity and the incidence of MODS in traumatized children.

## Background

Trauma is known as leading cause of morbidity and mortality in children older than one year of age [1,2]. Suffering from severe injuries, children commonly sustain blunt trauma mechanisms emphasizing on falls as well as pedestrian and cyclist motor vehicle impacts [3]. Due to different anatomic characteristics compared to adults [4], injury pattern differs sustaining especially life threatening head, chest and abdominal injuries [5]. Despite this, children suffering from multiple injuries have an improved survival as compared to adults [6]. Nevertheless, although the incidence of multiple organ failure (MODS) in pediatric trauma patients is lower than in the adult population [7] an overall mortality of 54% in pediatric MODS due to various reasons was described [8,9]. Furthermore, children appear to develop MODS in a different temporal pattern than adults: In children, MODS usually develops rapidly after ICU admission often within the first 4 to 7 days [9-11] suggesting a relevant influence of the innate immune response to trauma [10]. However, in adults, major trauma initiates a two-fold compromise of the immune system with hyperinflammation in response to injury and subsequent immunosupression [11,12]. Posttraumatic hyperinflammation is characterized by local and systemic release of pro-inflammatory cytokines, metabolites and acute phase proteins [13] leading to a systemic inflammatory response syndrome (SIRS) [13,14]. Later, anti-inflammatory mediators are released inducing immunosupression with susceptibility to infection and septic complications during the further clinical course [15]. The imbalance of this dual immune response seems to be responsible for organ dysfunction and multiple organ failure in adults [13]. In order to evaluate the severity of trauma and the risk for developing multiple organ failure, the pathophysiology of trauma was analyzed to determine potential prognostic markers [13,16]. Lately, Interleukin (IL-)6 as part of the pro-inflammatory cascade was verified as the most reliable prognostic marker [17]. IL-6 correlates with injury severity [18] as well as the incidence of multiple organ failure and outcome [17,19].

While most of the studies were conducted in adults, clinical data regarding the significance of cytokine levels for pediatric trauma still do not exist [10]. Despite the suggestion that the inflammatory response to major injury in children is functionally unique [7,10], prognostic markers predicting pediatric multiple organ failure are missing. We therefore intended to verify whether IL-6 plays a pivotal role in pediatric trauma similar to traumatized adults.

## Methods

The present study follows the guidelines of the revised UN declaration of Helsinki in 1975 and its latest amendment in 2008 (6th revision). The present study was approved by the institutional ethical review board. No informed consent was required. The study was performed at the Hannover Medical School, Hannover, Germany.

### Setting and patients

Traumatized children primarily admitted to a level I trauma center during a four-year period between January 1^st^ 2005 and December 31^st^ 2008 were eligible for the present prospective study if the following inclusion criteria were fulfilled: age less than 18 years, Injury Severity Score > 9 points [20,21], and consecutive admission to the hospital’s pediatric intensive care unit. The data were raised after inclusion by two independent reviewers (MF and FH).

### Demographics, injury severity and clinical course

Age and gender were measured as demographic parameters. The overall injury severity was classified by the established Injury Severity Score (ISS) basing upon the Abbreviated Injury Scale (AIS, latest revision 2005), determining the severity of individual injuries [22]. Clinical course included duration of ventilation (hours), duration of ICU treatment (days) and the overall length of stay (LOS) in days.

### Multiple organ dysfunction syndrome and outcome

Organ function status was evaluated according to the established score described by Marshall et al. [23] for at least 14 days after hospital admission. In brief, five organ dysfunctions (lung, liver, renal, hemodynamic and consciousness) were scored daily from nought (no dysfunction) to four points (severe dysfunction). Failure of organ function was considered at three or more points [23]; MODS was defined as simultaneous failure of at least two organs [8]. Children with development of MODS were assigned to group I and with uneventful clinical course to group II.

Primary outcome was defined as MODS during clinical course. Furthermore, mortality rate was evaluated by this study.

### Inflammatory marker

IL-6 [pg/ml] was measured with blood samples taken during stabilization in the emergency department after hospital admission. These samples represent the immunological response to trauma before emergency surgery was initiated (day 1). During the intensive care treatment, blood samples were taken repetitively every morning at 07:00 a.m. for at least 14 days. The IL-6 samples were part of the daily routine performed at the Center of Laboratory Medicine, Hannover Medical School, Hannover, Germany. Therefore, there was no need for special blood collection for this study. The measurement of IL-6 was performed with the IMMULITE 2000 XPi Immunoassay System©, Siemens AG©, Healthcare Sector, Erlangen, Germany.

### Statistics

The data were analyzed using the Statistical Package for the Social Sciences (SPSS; version 22; IBM Inc., Somers, NY, USA).

Incidences are presented with counts and percentages while continuous values are presented as arithmetic mean and standard deviation (SD). IL-6 levels are presented as median with 25- and 75-interquartile ranges (IQR 25 - 75). Apriori, Kolmogorow-Smirnow-Test was used to evaluate whether the variables were distributed normally. Differences between the groups were evaluated with Student’s t-test (ISS) or Wilcoxon rank sum test (Age, AIS distribution, treatment duration, IL-6 levels) for continuous data, while Pearson’s χ2-test was used for categorical variables. The Spearman rank correlation coefficient was used to determine the connection between IL-6 levels and the injury severity as well as the development of MODS. In addition, Pearson’s correlation was used to evaluate the linear relationship between these variables. Finally, a “Receiver Operating Characteristic” (ROC) analysis was performed to calculate the sensitivity and specificity of IL-6 towards the incidence MODS.

A two sided p-value < 0.05 was considered to be significant.

## Results

### Demographic data

In total, 59 traumatized children with a mean ISS of 23.9 ± 11.9 points were treated during the study period. The mean age for all patients was 8.4 ± 4.4 years, and 57.6% were male (n = 34). Referring to the complication of interest, 11.9% children (n = 7) developed MODS. Overall mortality was 3.4% (n = 2) due to multiple organ failure.

### Multiple organ dysfunction syndrome and outcome

No differences were found referring to age, gender, injury distribution or overall injury severity between children with and without development of MODS (Table 1). However, development of MODS lead to increased duration of ventilation, intensive care treatment as well as the overall length of stay (Table 1). Mortality was only found in patients with MODS (n = 2).

**Table 1:** Demographic parameters, injury distribution and injury severity compared between children with development of MODS and those without MODS

	**Children with MODS**	**Children without MODS**	**p-value**
	**Group I**	**Group II**	
**Number of patients % (n)**	**11.9% (7)**	**88.1% (52)**	-
Age (years)			0.847
Mean ± SD	8.7 ± 4.7	8.4 ± 4.4	
Median [IQR 25-75]	7 [6-13]	9 [5-12]	
Gender (♂ : ♀)	5 : 2	29 : 23	0.431
AIS head			0.818
Mean ± SD	2.9 ± 2.7	2.6 ± 1.8	
Median [IQR 25-75]	5 [0-5]	4 [0-5]	
AIS chest			0.158
Mean ± SD	2.3 ± 1.3	1.5 ± 1.7	
Median [IQR 25-75]	2 [2-3]	2 [1-3]	
AIS abdomen			0.534
Mean ± SD	1.4 ± 1.8	1.1 ± 1.4	
Median [IQR 25-75]	0 [0-3]	0 [0-2]	
AIS extremities			0.935
Mean ± SD	1.0 ± 1.2	1.0 ± 1.2	
Median [IQR 25-75]	1 [0-2]	1 [0-2]	
ISS			0.227
Mean ± SD	29.0 ± 7.4	23.3 ± 12.3	
Median [IQR 25-75]	29 [25-38]	24 [13-29]	
Duration of ventilation (hours)			0.001
Mean ± SD	418.7 ± 584.7	99.7 ± 133.5	
Median [IQR 25-75]	249 [90-344]	27 [0-183]	
Intensive care treatment (days)			0.003
Mean ± SD	21.1 ± 22.2	7.9 ± 8.3	
Median [IQR 25-75]	15 [7-21]	5 [2-11]	
Overall length of stay (days)			0.010
Mean ± SD	27.0 ± 21.8	14.3 ± 10.1	
Median [IQR 25-75]	19 [10-41]	12 [8-19]	
Mortality % (n)	28.6% (2)	0.0% (0)	-

### Inflammatory marker

**Figure 1:**
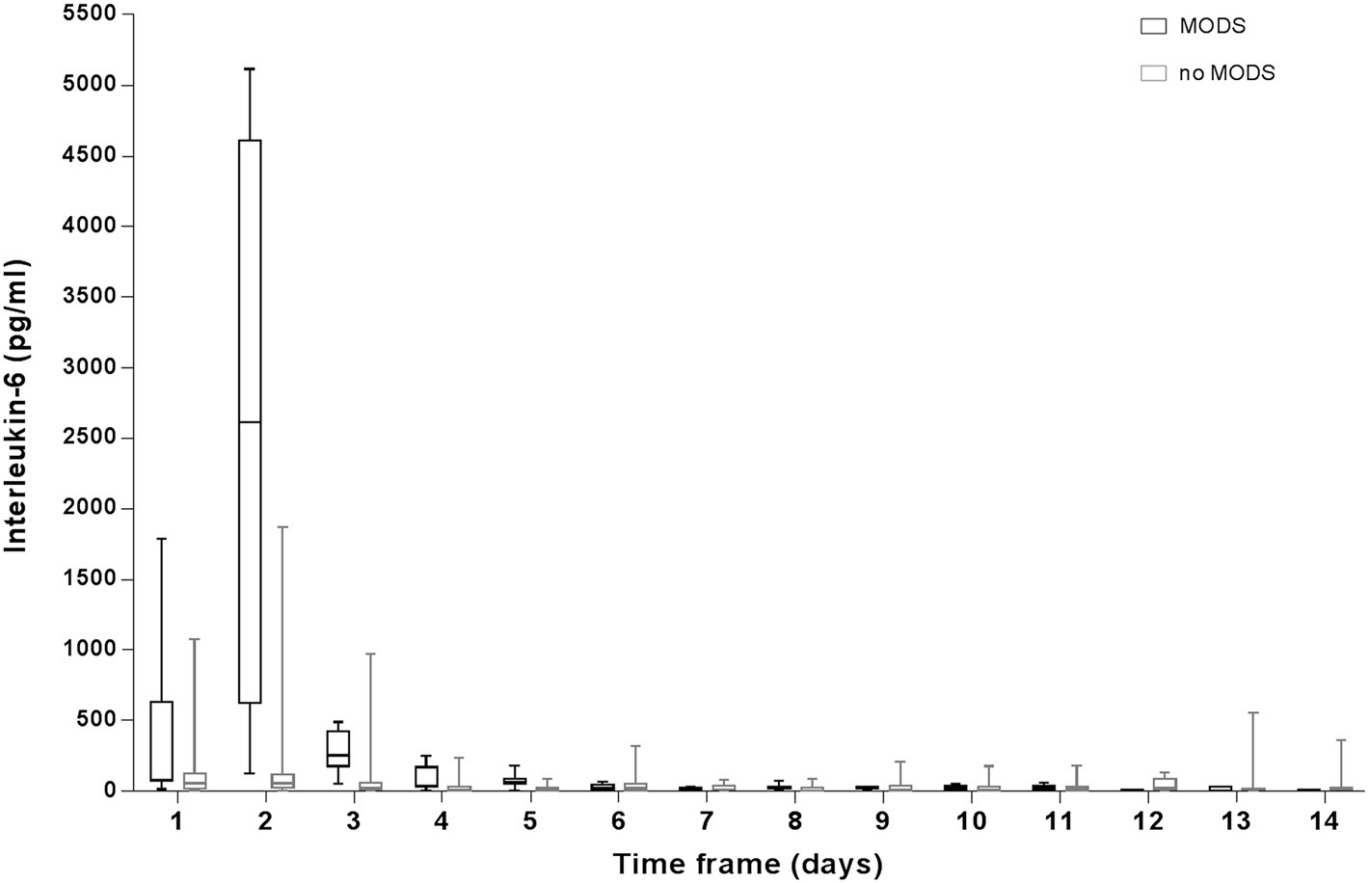
**The course of IL-6 comparing children with and without MODS.** Legend: abscissa: days. ordinate: IL-6 pg/ml. Significant differences were found at day 1 and 2 between the groups.

The descriptive course of systemic IL-6 levels comparing children with MODS versus those with uneventful clinical course is illustrated in Figure 1. In summary, traumatized children with development of MODS have been admitted with an increased mean IL-6 compared to children with uneventful healing (group I: 86 pg/ml [IQR 73-633 pg/ml] vs. group II: 57 pg/ml [IQR 20-125 pg/ml]; p = 0.045) (Figure 1). On day two, plasma IL-6 levels were increased in children developing MODS (group I: 2614 pg/ml [IQR 624-4609 pg/ml] vs. group II: 57 pg/ml [IQR 25-118 pg/ml]; p < 0.001). After a considerable decrease on day three (group I: 252 pg/ml [IQR 177-424 pg/ml] vs. group II: 23 pg/ml [IQR 11-64 pg/ml]; p = 0.054), no further differences could be measured for the further clinical course (p > 0.05).

Emphasizing on the potential prognostic value of IL-6, the evaluated IL-6 levels were correlated with the injury severity and the incidence of MODS (Table 2). While increased IL-6 levels during hospital admission were associated with the injury severity (r = 0.522, p < 0.001), any association to the incidence of MODS could not be shown at this time (r = 0.180, p = 0.231). However, increased IL-6 levels on the following two days were no longer associated with injury severity (day 2: r = 0.253, p = 0.106; day 3: r = 0.061, p = 0.718) but a significant correlation to the incidence of MODS was present on day 2 and 3 (day 2: r = 0.445, p = 0.003; day 3: r = 0.437, p = 0.007). According to Pearson’s correlation demonstrated in Table 3 a slightly different result is presented with increased IL-6 values on admission being correlated to MODS as well.

**Table 2:** Correlation of systemic plasma IL-6 values with injury severity and the incidence of MODS during the clinical course

	**Correlation coefficient**	**Correlation coefficient**
	**Injury severity (ISS)**	**MODS incidence**
**Day 1 (Admission)**	0.522*	0.180
**Day 2**	0.253	0.445*
**Day 3**	0.061	0.437*
**Day 4**	-0.126	0.295
**Day 5**	0.001	0.495**
**Day 6**	-0.156	0.047
**Day 7**	-0.123	0.043
**Day 8**	0.081	0.296
**Day 9**	0.189	0.210
**Day 10**	-0.172	0.112
**Day 11**	0.002	0.035
**Day 12**	0.334	-0.494
**Day 13**	0.227	-0.323
**Day 14**	0.521	-0.829

(Spearman rank correlation; *p < 0.01, **p < 0.05).

**Table 3:** Correlation of systemic plasma IL-6 values with injury severity and the incidence of MODS during the clinical course

	**Correlation coefficient**	**Correlation coefficient**
	**Injury severity (ISS)**	**MODS incidence**
**Day 1 (Admission)**	0.331*	0.297**
**Day 2**	0.115	0.717*
**Day 3**	-0.069	0.320
**Day 4**	-0.170	0.284
**Day 5**	-0.041	0.598*
**Day 6**	-0.107	-0.097
**Day 7**	-0.181	-0.196
**Day 8**	0.135	0.206
**Day 9**	0.196	-0.085
**Day 10**	0.015	-0.122
**Day 11**	0.112	-0.089
**Day 12**	0.192	-0.410
**Day 13**	0.124	-0.197
**Day 14**	0.195	-0.301

(Pearson correlation; *p < 0.01, **p < 0.05).

**Figure 2:**
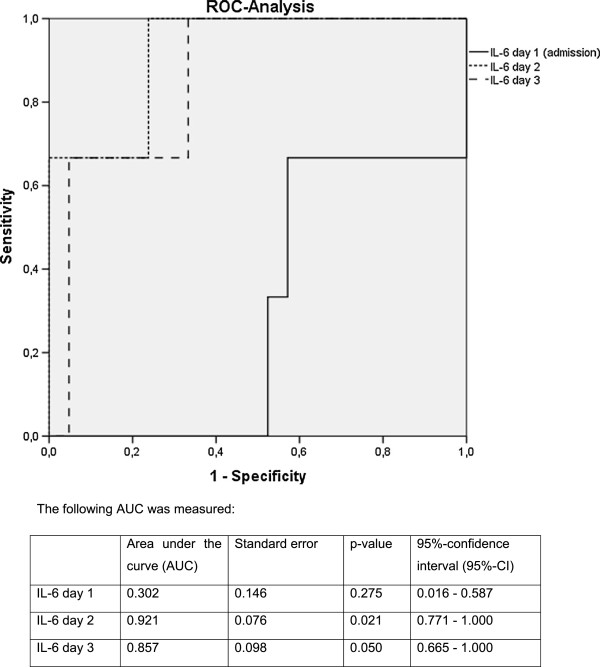
ROC-analysis of IL-6 towards MODS on the first three days.

According to ROC analysis IL-6 values on day 2 presented an area under the curve (AUC) of 0.921 towards MODS (p = 0.021) (Figure 2).

## Discussion

The incidence of post-injury multiple organ failure in traumatized children has been described lower than in adults [7]. However, this considerable clinical complication leads to a consequent mortality up to 50% in children as well [8]. Based on epidemiological studies regarding the incidence of pediatric multiple organ failure differences in the inflammatory response to trauma in children compared to adults can be suggested [9]. Nevertheless, clinical laboratory data of cytokine levels for pediatric trauma are still lacking [10]. Therefore, we intended to analyze the established marker IL-6 in severely traumatized children in order to verify if considerable coherences similar to traumatized adults exist.

The results of the present study could be summarized as follows:

11.9% of included children developed MODS with a subsequent mortality of 28.6%.

No influence of age, gender, injury distribution or injury severity could be shown. Children with development of MODS had significantly increased IL-6 levels during the first two days. Increased IL-6 levels during hospital admission were associated with injury severity, while an inconsistent association towards the development of MODS was proven at hospital admission. Increased IL-6 levels on day 2 and 3 were no longer associated with injury severity, but a correlation to the development of MODS was present.

The epidemiology of MODS in children has been described in various clinical settings including children with sepsis, congenital heart diseases as well as organ and bone marrow transplantations [8,9,11,24-26]. According to these settings, an incidence of MODS between 4% and 90% was demonstrated [8,9,11,24-26]. However, data about incidences of MODS in severely traumatized children are very sparse with only one clinical trial by Calkins et al. [7]. Retrospectively, the authors found only 3% of their severely traumatized children developing MODS. The authors suggested that traumatized children might be protected from sustaining this considerable complication compared to adults. Speculations were made if the lower incidence may be based upon a different immune response to trauma or if the hormonal milieu of children influences MODS and subsequently mortality. The interested and critical readership responded that children have closer monitoring of their fluid resuscitation and thus less fluid overload-induced lung injury determined the low incidence of MODS [27]. Furthermore, injury distributions as well as co-morbidities in the adult counterpart were listed as potential reasons for the decreased incidences of MODS [28]. However, the lack of current research leaves space for speculations about the reduced incidence of MODS in traumatized children.

Nevertheless, the detailed analysis of the immune response to trauma in adults [13] suggests that immune mediators might play a pivotal role in traumatized children as well [10]. This hypothesis is supported by findings of Zingarelli et al., who validated an age-related susceptibility for hemorrhagic shock-induced acute lung injury and SIRS in a rat model [29]. Systemic and end-organ manifestations of uncontrolled inflammation were regulated by peroxisome proliferator-activated receptor gamma. Regulation of this receptor was age-related attenuating the inflammatory response if administered in young but without effect in mature rats. Furthermore, in a clinical study, circulating cytokines in adults and children with burn injury were measured [30]. Systemic levels of several mediators, e.g. IFN-γ, IL-10, IL-17, IL-4, IL-6, and IL-8, were increased in adults compared to children [30]. The authors concluded that discrepancies between children and adults suggest age-dependent therapeutic interventions to achieve attenuation of the inflammatory response. As demonstrated by Finnerty et al., various laboratory parameters can be applied including pro- and anti-inflammatory parameters [30]. Furthermore, alarmins have been demonstrated to be involved in the early immune response after trauma [31]. These include heat shock proteins, annexins, defensins, and the high mobility group Box 1 (HMGB1) [31]. Especially HMGB1 is currently in the focus regarding the very early stage of triggering a sterile inflammation within 30 minutes after trauma [31]. In this respect, HMGB1 is stimulating macrophages and endothelial cells to release TNF-alpha, IL-1 and IL-6 [31]. Arguing on daily clinical practice, IL-6 has been the most useful and widely employed mediator because of its plasma half life and relatively consistent release pattern in posttraumatic inflammatory research [32]. Consequently, we intended to evaluate the established IL-6 in severely traumatized children: The present study shows a considerable correlation between IL-6 levels and development of multiple organ failure after trauma in children confirming results from other investigations in adults [17,32,33]: In a prospective study analyzing 75 severely injured adult patients, plasma IL-6 levels were increased in MODS patients during the first 10 days [33]. In contrast to the present study, the highest measured values were noted on admission with a continuous decrease during the following ten days. Furthermore, Giannoudis et al. elucidated increased levels of IL-6 in severely injured patients on the first five days in the presence of MODS [32]. With an increase of plasma IL-6 levels on the first day after admission and a subsequent decrease on the following days, the descriptive IL-6 course in adults with MODS was similar to the results of the present study. However, emphasizing on the quantitative levels of IL-6, we found increased levels (hospital admission: mean IL-6 2,616.3 pg/ml) in our pediatric population compared to the aforementioned studies in adults (mean IL-6 1,000 pg/ml) [17,32]. Surprisingly, this ratio is in contrast to the inflammatory reaction described after burn injury showing increased levels in adult patients [30]. In accordance with our results, children with isolated traumatic brain injury showed significantly increased plasma IL-6 levels [34] compared with adults [35]. Moreover, in the early post-traumatic period (first 24 hours after head injury), the systemic levels of IL-6 appeared to be increasing in pediatric patients similar to findings in our study [34]. This phenomenon was particularly evident in those children with worse outcome [34]. Consequently, one might conclude that the inflammatory reaction to blunt trauma differs as compared to burn injuries. This suggestion is supported by an immunological study evaluating pediatric and adult immune responses to infectious impacts [36]: No differences between pediatric and adult interleukin levels were provided although correlations to worse outcome were proven [36]. Based on these results we suggest that the pediatric immune response to trauma seems more sensitive compared to adults resulting in increased cytokine levels. Interestingly, the measured correlation of IL-6 and the incidence of MODS on the second and third day is congruent to the elucidated prognostic time frame in adults: With an increase of IL-6 during the first days, some authors stated that the prognostic period of IL-6 ends after three days, indicating that IL-6 levels have no prognostic value after that time point [33,37]. However, since pediatric trauma leads to increased plasma IL-6 levels but has a lower incidence of MODS as compared to adults, increased cytokine levels do not necessarily determine an increased incidence MODS. One might hypothesize that the cellular reaction and consequent cytotoxic effect to the inflammatory impact could be less severe than in adults. Further investigations of the cellular and molecular mechanisms in the posttraumatic pediatric inflammatory response are necessary to elucidate the role of cytokines and related systemic consequences. Especially research on complement and alarmin trigger mechanisms could potentially reveal a different endogenous mechanism of stimulation of macrophages and endothelial cells in pediatric trauma patients. Although the measurement of HMGB1 and toll-like receptors as initiators and modulators of cytokine release seems impracticable for daily clinical routine these parameters could help to understand the dynamic of pediatric inflammatory response after trauma.

Beside the discussed association of IL-6 levels towards the incidence of MODS, IL-6 is commonly known to describe the severity of trauma as well. Gebhard and colleagues evaluated plasma IL-6 levels hourly after severe trauma in 94 traumatized adult patients [18]. Their results revealed an early increase immediately after trauma. Overall, the best correlation of IL-6 release with the ISS values was described during the first 6 hours after hospital admission which seems congruent with our results. In addition, Strecker et al. prospectively evaluated 107 traumatized adult patients collecting blood samples on admission until the 10^th^ day after trauma [38]. Similar to the presented results, correlation between systemic plasma IL-6 levels and the ISS succeeded only within the first 24 hours after admission. However, the presented study is the first demonstrating a correlation of increased IL-6 values with the extent of trauma in children. Similar to previous results in adult patients, increased IL-6 values display the extent of trauma only in the early period.

The presented analysis has several notable limitations. Critics to the presented study could be remarked due to the fact that the MODS score used has not been validated in children. Yet, other commonly used scoring systems have the same limitations [39,40]. Referring to Calkins et al. [7], the currently established pediatric scoring systems [41,42] do not differentiate between previously healthy trauma patients and children with life threatening chronic diseases. Thus, pediatric organ dysfunction scoring in traumatized patients remains to be defined [7]. According to the presented results, support of organ function, e.g. ventilation support and dialysis, was performed by the treating ICU team in MODS patients as defined in our study. Apriori, the small number of patients limits the statistical power although this number seems comparable to prospective studies in adults [17,33]. However, the incidence of pediatric patients suffering from major trauma is rather low; thus, multi-center studies with other limitations are required to reasonably increase patients’ number. Referring to the inclusion criteria of an ISS > 9 points we feel safe to describe our study population severely injured due to the measured mean ISS of approximately 24 points which is comparable to the study population by Calkins et al., who demonstrated a mean ISS of 26 points despite the inclusion criteria of ISS >15 [7]. However, the most considerable limitation is the lack of a comparative adult trauma population. In order to enable direct comparison between children and adults, further research in similar settings is needed to guarantee an adequate perception.

Despite these limitations, this article does elucidate that IL-6 after pediatric trauma is associated with an increased risk of MODS.

## Conclusions

The relationship between clinical and laboratory evaluation of systemic inflammation has been described in adult trauma patients evaluating IL-6 as most reliable marker. The presented prospective study is now the first providing evidence of a correlation of systemic IL-6 levels with injury severity and the incidence of MODS in pediatric trauma patients. Further studies are required to elucidate prognostic levels of IL-6 and to determine the effect of inflammatory mediators following trauma in children.

## Ethical review committee statement

This study followed the guidelines of the revised UN declaration of Helsinki in 1975 and its latest amendment in 2008 (6th revision). The study was approved by the institutional ethical review board.

## Competing interests

This study was funded by a grant of TUI-Stiftung, Hannover to M. Frink and F. Hildebrand. The authors declare that they have no competing interests.

## Authors’ contributions

HA conceived this study designing the trial, provided statistical advice on study design, analyzed the data and drafted the manuscript. JF, UB, AG, JHKA and MS provided statistical advice on the study design, analyzed the data and supervised the conduct of the trial and data collection. MS, AG, JF, UB and JHKA conceived the study and designed the trial. MF conceived the study, designed the trial, obtained research funding and supervised the conduct of the trial. FH conceived the study, designed the trial, obtained research funding, supervised the conduct of the trial and data collection, provided statistical advice on study design and analyzed the data. MF takes responsibility for the article as a whole. All authors contributed substantially to manuscript revision. All authors have read and approved the final manuscript for publication.
